# Contextualizing adolescents’ self-awareness of problematic mobile phone use: a preliminary study

**DOI:** 10.12688/f1000research.51339.2

**Published:** 2021-08-23

**Authors:** Andrew Karnaze, Katherine Grevelding, Traci Marquis-Eydman, Douglas McHugh

**Affiliations:** 1 Frank H. Netter MD School of Medicine, Quinnipiac University, North Haven, CT, 06473, USA; 2 School of Health Sciences, Quinnipiac University, North Haven, CT, 06473, USA

**Keywords:** Public Health, Health Behavior, Adolescents, Problematic Smartphone Use, Mobile Phones, Cell Phones, Social Media, Qualitative Methods

## Abstract

Adolescents engage cognitively, emotionally, and behaviorally with smartphones. Growing evidence suggests they struggle to interact with them in moderation, which has been framed in relation to behavioral addiction as problematic mobile phone use. This study contextualized 13-15 year-old adolescents’ self-awareness of problematic mobile phone use. Focus groups were conducted with 11 adolescents who assessed themselves using the problematic use of mobile phones scale. The authors used interpretative phenomenological epistemology as a guiding framework. Audio recordings were analyzed qualitatively using a constant comparison approach. Students agreed or strongly agreed with multiple dimensions of the problematic mobile phone use construct. Four major themes emerged in relation to circumstances, factors, processes, constraints, and opportunities: *drivers of excessive smartphone use*, *with family or friends*, *barriers to healthier smartphone use*, and *nighttime habits.* Adolescents’ assessment of perceived proper versus problematic mobile phone can inform hypotheses targeted at improving overall wellness and developing healthy habits in adolescence that carry over into young adulthood and beyond.

## Introduction

Following the release of the iPhone in 2007, estimated smartphone ownership in the United States has steadily increased to 81% in 2019 and 96% for 18-29 year-olds ( www.pewresearch.org/internet/fact-sheet/mobile/#mobile-phone-ownership-over-time). Mobile phone dependency (a.k.a. problematic mobile phone use) has grown prevalent in keeping with this surge, ^1– 2^ and has been framed in relation to behavioral addiction. ^3^ For the purposes of this study, we used the definition “problematic mobile phone use is a habitual drive or compulsion to continue to repeat a human-technology interaction despite its negative impact on one’s well-being.” ^3^ Among the general population, determinants and effects of problematic phone use have been studied, screening tools developed, and prevalence of mobile dependency measured. ^2, 4– 5^ While the detrimental effects of problematic mobile phone use on sleep, stress, anxiety, and depression can occur at various ages, ^2^ adolescents comprise a notably vulnerable age group. This relates to decreased levels of self-control and low resistance to peer pressure. Steinberg and Monahan (2007) noted “susceptibility to peer pressure in adolescence follows an inverted U-shaped curve, increasing during early adolescence, peaking around age 14, and declining thereafter.” ^6^

We searched the PubMed database using the query “((Smartphone OR mobile phone OR cell phone) AND (dependency OR dependence OR problematic)) AND (adolescent OR adolescence OR teenage OR youth)” and found with regard to adolescents that studies are limited to reports of prevalence and associations with adverse wellness. More specifically: cross-sectional studies have measured prevalence of mobile phone dependency ^1, 7– 8^; mobile phone dependence correlated positively with unintentional injuries ( *odds ratio* = 1.452) ^9^; depressive symptoms, higher interpersonal anxiety, and lower self-esteem have been correlated with problematic cell phone use ^10– 12^; and Walsh *et al* (2008) held qualitative focus groups with Australian 16-24 year-olds and found extreme attachment to phones with symptoms of behavioral addiction evident in participants' descriptions. ^13^ Adolescent-specific investigations are limited to mobile phone dependency prevalence ^1, 7^; and to correlation with depression, interpersonal anxiety, lower self-esteem, and symptoms of behavioral addiction evident. ^10, 13^ Adolescents are knowledgeable agents capable of representing and articulating the different aspects of how they interact with smartphones. Yet little work has been devoted to exploring how adolescents experience mobile phone dependency and what meanings and interpretations they attach to it.

This preliminary study sought to contextualize 13-15 year-old adolescents’ self-awareness of problematic mobile phone use by exploring interconnections between mobile phone dependency phenomena, the context in which they occur ( *i.e.*, circumstances, factors, processes, constraints, or opportunities), and participants’ subjective experiences and interpretations. We believe this to be a crucial step towards generating hypotheses targeted at improving overall wellness and developing healthy habits in adolescence that can carry over into young adulthood and beyond.

## Methods

Quinnipiac University’s Institutional Review Board approved this study (protocol #01018; 9 February 2018).

### Recruitment and Consent

Eleven 13-15 year-old adolescents were recruited by convenience sampling to participate: six from Morgan High School (School #1; five females, one male) in Clinton, CT, USA and five from Dodd Middle School (School #2; three females, two male) in Cheshire, CT, USA. Email invitations were sent to eligible participants by School Guidance Counsellors. Inclusion criteria: age 13-15; uses a smartphone; speaks English; and parental consent was obtained. No participants dropped out of the study.

### Research Approach

This study was conducted through a constructivist lens using an interpretative phenomenological epistemology. ^14^ We chose a phenomenological approach to better understand participants’ interpretation of the contexts in which they interact with their smartphones. Phenomenological study is a qualitative research method that is used to describe how human beings experience a certain phenomenon and make meaning out of it. Researchers analyze the perceptions, perspectives, understandings, and feelings of those people who have experienced or lived the phenomenon. ^15^ Thus, phenomenology may help us learn from others’ experiences by describing what was experienced and how it was experienced. ^14^ We selected a constructivist lens to align with our phenomenological methodology based on the assumption that internal and external constructs influence an individuals’ knowledge and experience. ^16^

### Data Collection and Analysis

In order to establish evidence of mobile phone dependency, participants assessed themselves using hardcopies of the problematic use of mobile phones (PUMP) survey. ^29^ Merlo et al. (2013) developed this 20-item instrument as a self-reported measure of the 10-dimension construct *problematic mobile phone use*, based upon the diagnostic and statistical manual of mental disorders 4 ^th^ edition (DSM-IV) criteria for substance use disorders. ^4^ They published validity evidence supporting PUMP’s use with English speakers; namely it demonstrated: 1) a single-factor structure via principal components analysis; 2) excellent internal consistency (Cronbach α = 0.94); 3) convergent validity when compared to the existing cellular phone dependence tendency questionnaire, cell phone usage data (duration and frequency measurements), and self-perceptions of addiction; and 4) discriminant validity when compared to length of time a phone has been owned and money spent monthly on mobile phone minutes. ^4^

Author K.G., facilitated the focus group sessions; she is an assistant professor with an educational doctorate (EdD) and expertise in qualitative research methodology and focus group facilitation. K.G. had no established relationship with any of the participants prior to study commencement. Participants knew about K.G.’s qualifications and the general purpose of the research study from the informed consent documents. Two 90-minute focus groups were held, one for the Clinton High School cohort on June 18, 2018 and one for the Dodd Middle School cohort on July 12, 2018, in a reservable meeting room of the Henry Carter Hull Library in Clinton, CT. Author A.K. was present as a non-participatory observer. They began with 60 minutes of facilitated, semi-structured discussion, then participants watched an eight-minute video clip on social media app development before discussion resumed ( CBSNews.com. Brain Hacking. www.youtube.com/watch?v=awAMTQZmvPE&t=1s time: 0-5:30, 8:45-11:30). This video clip artifact was employed to help elicit participants’ views on *Snapstreaks.* It is not uncommon in humanities or sociology qualitative research for researchers to employ the use of artifacts (i.e., something created by humans which gives information) for participants to react or respond to. ^17^^,^^18^ These artifacts are used, along with an episodic semi-structured interviewing style, to elicit participants’ views about the subject under investigation. The primary goal of researchers during the data analysis process in a phenomenological study is to deduce meaning out of the themes identified through organizing the data, coding the data, deducing categories, identifying common themes, making interpretations, and maintaining a reflective journal. The latter is an important step on phenomenological study to address researcher bias and overall credibility. It involves maintaining field notes detailing researchers’ choices and decision-making; it serves to keep the themes generation and interpretation transparent, coherent, and simple. ^19^

K.G. wrote field notes after each focus group to reflect on the participants’ observations, K.G.’s growing insights, and how her facilitator-outsider role informed the research process. ^20^ Audio recordings were transcribed verbatim then subject to de-identified inductive qualitative analysis using a constant comparison approach. ^21^ De-identified transcripts were read, then hand-coded by assigning first-stage preliminary codes. A.K. and D.M. then met together to group similar codes into categories and identify major concepts while referencing K.G.’s memos and field notes. Iteratively, major concepts were further organized and expressed as themes and subthemes.

## Results

### PUMP Survey

**Figure 1.**
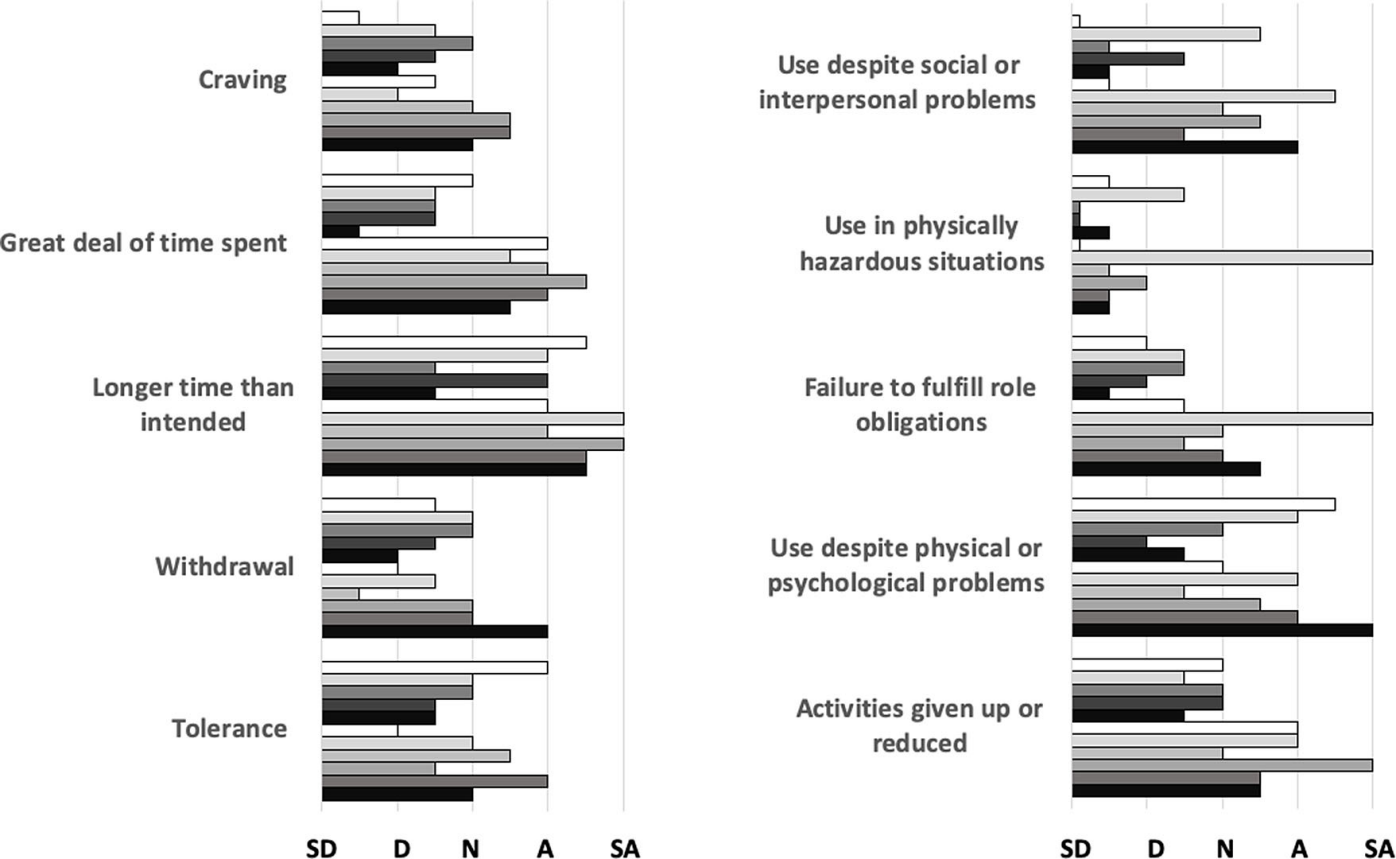
Frequency distribution of responses on a 5-point scale ( *strongly disagree, disagree, neutral, agree, strongly agree*) to the 10 dimensions of the PUMP survey; each bar represents one of eleven individual participants.

One or more participants agreed or strongly agreed with items representing nine of the ten dimensions of the PUMP construct. *Longer than intended*, *great deal of time spent*, *activities given up or reduced*, and *use despite physical or psychological problems* were most frequently agreed with; *use in physically hazardous situations*, *withdrawal*, and *craving* least frequently ( Figure 1).

### Qualitative Findings

Phenomenological data explication involves researchers’ inferring the meaning behind participants’ words and articulating experiences through thematic statements. ^22^ Participants possessed a notable awareness of problematic smartphone use in themselves and their peers. Four main themes with associated subthemes emerged from our phenomenological data analysis processes: *drivers of excessive smartphone use*, *with family or friends*, *barriers to healthier smartphone use*, and *nighttime habits*; Illustrative quotes are identified by anonymous participant codes. All perspectives were shared by both female and male participants alike with the exception of the sleepover comments for theme 2A, which were limited to female participants.

### Theme 1: Drivers of excessive smartphone use

There was consensus among participants that a few social media apps ( *e.g.*, Instagram, Snapchat) and texting were among the predominant drivers of excessive smartphone use. They or others had observed they were spending a great deal of time, or longer than intended, interacting with their smartphone, which kept them from other important work ( *e.g.*, schoolwork). Participants interacted and re-interacted with these apps habitually, even when there was no longer unknown, new, or updated content. Notifications ( *i.e.*, visual, audible, or tactile alerts) bolstered excessive use by prompting phone-checking and re-prioritization of current activities. Delaying or avoiding unpleasant tasks or managing stress were also connected with extended phone use.

*Theme 1A: A few smartphone apps take up the majority of adolescents’ time*

“I’ll have lots of apps on my phone, but really it’s two or three that I’ll actually be using. And those apps alone are just driving the time up … To just be scrolling through Instagram, to just be on, just texting non-stop. And that’s not really a good habit.” ( *School #1 student C, female, 15*)

“Specifically with Snapchat, I remember deleting it for an amount of time and I felt really, I was never on my phone and it was all because of this one app.” ( *School #2 student B, female, 13*)

*Theme 1B: Reiterative use of apps despite an awareness of the lack of fresh content*

“I don’t even have a reason to go on my phone. I just go on it, even if nothing’s new and it’s like five seconds later, I just switch between [social media] apps and even if it’s completely the same as I saw five seconds ago.” ( *School #2 student C, female, 15*)

“I’m aware most of the time I’m not gaining anything from using it.” ( *School #2 student A, male 15*).

*Theme 1C: Notifications lead inevitably to checking of apps*

“I check my phone at least 50 times a day, at least … whenever I hear a ring on Snapchat or whatever, I’m like “Ooh, who’s texting me? It triggers me and I’m like “who’s texting me?, and then I have to check, right?” ( *School #2 student E, male 15*)

“I'll get 30 Snapchat in an hour or something … so I just turn the notifications off so I don't have that tendency to just keep checking and re-checking every time I see someone has sent me something.” ( *School #2 student D, female 13*)

“A lot of times I'll get notifications … then I just start thinking, ‘Oh, what if something is really important happened? I really need to know. Or, what if something changed in my schedule.’ If I don't look right now then that could impact my day.” ( *School #1 student D, female, 13*)

*Theme 1D: Phone interactions facilitate delaying or managing something unpleasant*

“Phone use is clearly a procrastination/dealing with stress tool.” ( *School #2 student A, male 15*)

“When there's a big test the next day and you come home from school … you don't wanna work at school and then come straight home and start working again. It's not what I do, at least. I take a little break, but sometimes I'm on my phone for a long period of time watching TV or on the internet for a long period of time. I'm procrastinating, procrastinating, procrastinating, making myself more stressed for this test that I still have to study for.” ( *School #2 student B, female, 13*)

### Theme 2: With family or friends

Participants were prepared to forego access to their phones in favor of spending quality time with close family, but not to de-prioritize phone interactions when gathered with close friends. Perceived hypocrisy with regard to family members’ phone use was also noted.

*Theme 2A: Phone interactions prioritized over friend interactions*

“I’ll be at a sleepover with one of my friends or like a group sleepover and you can look around the room and every single person’s on their phone and you’re all together. Most of the time, it's all the people that you talk to, and everyone's on their phone. Like, who are you talking to? Because all your friends are here. So, I don't know what's going on but that's causing everybody not to really talk and it's all just on your phone. People are just scrolling through Instagram or just going through random stuff, even though nobody has posted anything.” ( *School #2 student C, female, 15*)

I have noticed that sometimes I'll be with friends and I'll be literally Snapchatting them as they sit right next to me. And it makes no sense, but it's just a habit. I don't even know why, it just happens. ( *School #2 student B, female, 13*)

Walking around the school, people aren't even looking up from their phones to say hi to their friends. Or during lunch where it's quality time that you're supposed to [spend] talking to your friends, they use that time to go on their phones or just not talk to anyone next to them and be on Snapchat or Instagram. ( *School #2 student C, female, 15*)

*Theme 2B: Impacts quality time with family*

“I'm not allowed to use my phone when I'm on family vacations or spending time with my grandparents because my mom wants me to enjoy every moment that I have with them. It feels good. I just feel happier that I'm with my family, I'm not worried about who's texting me or what somebody's doing or anything like that.” ( *School #1 student F, male, 15*)

“My grandparents are getting old and they have Parkinson's and they're kind of deteriorating. And I'm on my phone and I feel so guilty because I'm like I only have 'em for a couple more years, so honestly, my texting other people can wait and stuff like that.” ( *School #2 student E, male, 15*)

*Theme 2C: Poor role-modeling*

“My mom always claims that I’m addicted. But then, so when I get off my phone, I always find that she’s on her phone or my sister’s on her phone … it might seem that it's just the kids, but it's also the adults at times, too.” ( *School #2 student D, female, 13*)

### Theme 3: Barriers to healthier smartphone use

When asked to identify obstacles that prevented them from reducing smartphone use, participants noted peer pressure, social media etiquette, and competitive approval seeking.

*Theme 3A: Peer pressure*

“It’s actually making people feel pressured … like you *have* to reply back. Gotta keep the [Snapchat] streak, gotta keep the friendship alive …” ( *School #2 student D, female, 13*)

“There's so much pressure to be on all the social media's. To be checking on people. If someone posts something, then everyone will start texting, ‘Look what this person posted.’ People saying, ‘You've got to join this app, you've got to join this app, you've got to get the newest phone.’ It's just so much pressure, and sometimes you just don't want to do that.” ( *School #1 student C, female, 15*)

*Theme 3B: Social media etiquette*

“I definitely think there’s an etiquette when it comes to social media, especially Instagram *.* A lot of the girls I know, if your best friend posts, you *have* to comment something on it.” ( *School #1 student D, female, 13*)

“I feel like there's kind of these rules. Just hidden little things that everyone needs to know about. Whoever gets the most likes, that's a big thing. ‘Oh, 200, 300 people liked this’. All the rules are different for every grade. No one really knows who started the rules, but one person would say something, and it'll get passed on. And pretty soon the whole grade will be following one rule or multiple rules about social media. And there's no real clear, this person spread it. It's just kind of just something that's taking form, and everyone should know, and everyone needs to adapt to essentially.” ( *School #1 student C, female, 15*)

*Theme 3C: Competition and approval seeking*

“Things become a competition, so when you’re on your phone you see someone post something and think, “Wow, that’s really cool, I have to do that, because that would make me so cool.” So then, everyone just keeps posting [their own version]. Trying to have the best post, and everyone like it. See who can get the most likes. It's just a big competition that it's not really a healthy environment to be in.” ( *School #1 student B, female, 15*)

### Theme 4: Nighttime Habits

Bedtime routines in relation to continued smartphone access arose in relation to sleep hygiene, wellness, and school.

*Theme 4A: Sleep Hygiene and Putting Phones Away*

“Going to sleep... the phone makes that harder, definitely … just talking to people especially on Snapchat, that can just extend your time before you go to sleep by a lot without you realizing it.” ( *School #2 student A, male, 15*)

“I've kind of gotten into the habit of I go to sleep at 9:00pm every night because I've been on my phone all day and I put it far away from my bed where I don't have the energy to get up and grab it. So, I put it there and it feels actually nice when I put it down because I feel like I'm just disconnected and I can be soothing and I don't have to worry about people texting me all the time. So, it feels pretty good.” ( *School #2 student C, female, 15*)

“There's other people at my school whose parents don't take away their phones at 10:00pm, so they'll be up to 2:00am or sometimes there's kids who are up all night, just on their phone for no reason. And that's super-unhealthy for their physical state when they're super-tired during school, they're not paying attention, they're not into it. They're sitting there falling asleep in school and they look terrible. They shouldn't be up all night on their phones. It's just unhealthy for them.” ( *School #2 student B, female, 13*)

## Discussion

Nine and six of the eleven participants agreed or strongly agreed with items representing the PUMP dimensions of *longer than intended* and *great deal of time spent*, respectively. We found that these phenomena occurred for our 13-15 year-olds when they were interacting with only a select number of social media or texting apps, and this was facilitated by habitual processes of evaluating and responding to notifications or checking and re-checking app content. This infers it is not the phone that drives problematic behavior, it is the apps. A significant reason that adolescents are attracted to social media apps is that they increase a sense of social inclusion and connectedness. ^8^ Social media tech companies have capitalized on this for the sake of monetization. They compete with one another for finite attention in a zero-sum game. One example of this is Snapchat, an app where users *snap* each other ( *i.e.*, send pictures or videos with overlying captions, emojis, or animations); as of March 2020, Snapchat had 229 million daily active users and 4 billion+ snaps are sent each day (Snap Inc. First Quarter 2020 Financial Results https://investor.snap.com/news-releases/2020/04-21-2020-210949737). A design feature called *Snapstreak* involves two users seeing how many days in a row they can maintain snapping each other. Snapstreaks are considered an indicator of success and are viewed by adolescents as proof of friendship (Lorenz T. 17 teens take us inside the world of Snapchat streaks, where friendships live or die. Mic. https://www.mic.com/articles/173998/17-teens-take-us-inside-the-world-of-snapchat-streaks-where-friendships-live-or-die). Adolescents are often sensitive and acutely aware of their own, and peers’, social dynamics; therefore, having tangible evidence of committed friendships that endure over time can be a meaningful source of acceptance and recognition. One of our participants shared, “I'll get 30 Snapchat in an hour or something”. Adolescents can be devasted if months- or year-long snapstreaks lapse that they have poured an immoderate amount of time, energy, and self-validation into. Tristan Harris has stated, “Snapstreak is driving some teenagers nuts—to the point that before going on vacation, they give friends their log-in information and beg them to snap in their stead” (Harris T. What’s Now San Francisco with Tristan Harris. https://youtu.be/YQh2FQ7MZdA). Harris refers to the persuasive tactics behind Snapstreaks as hijacking techniques intended to maximize viewership and consequently advertisement revenue. In addition to these conditions and processes, peer pressure, emergent social media etiquette, and competition for approval were factors that also fed into and shaped the relevant context here. These represent reinforcers; an operant conditioning term referring to anything that strengthens or increases a behavior. Our participants recognized the pressure to conform and do what their peers are doing to be powerful and hard to resist. This manifested in the form of coercion to maintain Snapstreaks and have an active presence with multiple prevailing social media apps. Social media etiquette, in setting conventions and expectations for behavior, connects to adolescents’ group identity. This reinforcer increased app engagement by threatening a person’s sense of belonging and how they feel about themselves in relation to their peer group. In this sense, creating and posting a distinct version of a notable social media post and competing with peers for ‘likes’ is normalized and offers raised perceived social status as a reward. Problematic smartphone use has been negatively associated with adolescents’ mental well-being. Souza Pereiva *et al*. (2020), ^23^ in a study of 359 female and 308 male students aged 13-18 years, associated it adversely with physical activity levels, personal interactions, academic performance, and mood disturbances. Abi-Jaoude *et al*. (2020) ^24^ also report, “Evidence from a variety of cross-sectional, longitudinal and empirical studies implicate smartphone and social media use in the increase in mental distress, self-injurious behaviour and suicidality among youth; there is a dose–response relationship, and the effects appear to be greatest among girls.” As we also noted from our participants’ responses, Abi-Jaoude *et al*. describe smart phone app-facilitated social comparisons among adolescents that negatively affected their self-view and led to increased cyberbullying and promotion of self-harming behaviors. They call for an increase in public awareness of problematic smartphone use. The contexts highlighted by our study may help inform social policy interactions aimed at promoting safe, inclusive, and nourishing home or educational environments.

Almost half of the participants agreed or strongly agreed with items representing the PUMP dimensions of *activities given up or reduced* and *use despite physical or psychological problems.* With regard to these phenomena, adolescents’ experiences were shaped by both constraints and opportunities. Problematic use of smartphones was limited when some participants were gathered socially with close family; they acceded to restrictions placed on their phone access. In contrast, when gathered for social activities with close friends, phone interactions were prioritized and quality time forfeited. Participants could not fully rationalize this behavior. Physical restrictions on the proximity of smartphones to adolescents’ beds at night was another relevant constraint that directly impacted sleep hygiene, wellness, and next day preparedness for education. Likewise, Smetaniuk (2014) studied 301 undergraduates and found that more than 20% deprived themselves of sleep due to late-night phone use. ^5^ Smartphone interactions provided adolescents with opportunities to delay or avoid something unpleasant ( *e.g.*, putting off studying for a test) or to manage stress. One of our participants noted, “You don't wanna work at school and then come straight home and start working again … I take a little break, but sometimes I'm on my phone for a long period of time … . I'm procrastinating, procrastinating, procrastinating, making myself more stressed for this test that I still have to study for.” This is consistent with the understanding that we mis-regulate ourselves by believing task avoidance will make us feel better. ^25^ By engaging with their smartphones as a coping mechanism, adolescents choose to focus on feeling good in the moment, in the now.

*Implications for Research and Practice*

The 21 ^st^ century has seen the convergence of widely accessible software apps, participatory media, and internet connectivity such that virtual learning and online coaching are no longer uncommon. Having reflected on our findings, we suggest that future studies explore the effectiveness of app-based digital coaching and management techniques to scaffold adjustments to adolescents’ habits in favor of a healthy phone-life balance. For example, Moment (Moment Health Inc., Burlingame CA) offers features suited to the contexts described here including goal-setting and guided coaching to: track phone and app usage, establish phone-free times with friends and family, stop sleeping with your phone, reduce distractions, role-model accountability, and stop aimless browsing. This could affect a novel approach to reducing mobile phone use that would place some of the impetus for change upon the adolescent and facilitate conversations between teens, parents, and care providers.

*Limitations*

This preliminary study was limited in that it involved only two adolescent cohorts from one geographical region of the United States. A minimum sample size of twelve participants is recommended for qualitative studies to reach data saturation (i.e., the point where redundancy signals to researchers that data collection may cease). ^26– 28^ Given this, our sample of eleven participants isn’t quite sufficient for us to assert that our qualitative data achieved thematic saturation. The timeline of recruitment, data collection, and data analysis for this study necessarily followed the availability of the first author A.K. while he was in medical school. The authors had intended to recruit a third group of adolescents in 2020 to increase the participant number above twelve but the SARS-CoV-2 coronavirus pandemic prevented this.

In addition, no cut-point(s) for the PUMP scale have been established, preventing us from making more concrete judgments about the *problematic mobile phone use* construct with regard to our participants.

## Conclusions

Smartphones have ensconced themselves into the everyday lives of consumers around the globe. The prevalence of problematic phone use amongst adolescents and its connection with adverse wellness is a growing public health concern because harmful behaviors established in childhood can shape one’s subsequent life course. Context is important for most phenomena of health care and health. This preliminary study supplies a contextualized glimpse into the adolescent assessment of perceived proper versus problematic smartphone use, potentially allowing parents, educators, and clinicians to reframe their approach to this critically important topic with the adolescents themselves.

## Ethics statement

This study was conducted according to the guidelines of the Declaration of Helsinki and approved by the Human Experimentation Committee/Institutional Review Board of Quinnipiac University (#01018; 9 February 2018).

## Consent statement

Written informed consent for publication of the participants’ details was obtained from the participants and parents/guardian/relative of the participant.

## Data availability statement

### Underlying data

The Quinnipiac University Institutional Review Board (IRB) required that the audio recordings of our focus groups not be made publicly available because 1) the participants were minors (adolescents 13-15 years old) and may accidentally use their real names rather than their assigned anonymous designations; e.g., “participant A” or “participant C” … etc and 2) that our small sample size increased the risk of them being identified if their voices were to be heard in conjunction with the names and locations of their Schools and dates of the study being publicly available. This requirement shaped our informed consent documents which stated that the original audio recording would not be made publicly available, but de-identified transcripts of the focus group sessions would be available upon request of the corresponding author/principle investigator.

Due to the age of the participants in this study, their parents/guardians did not agree for the original audio files to be shared publicly, so these data are not available to be shared. The de-identified focus group transcript data that support the findings of this study are available from the corresponding author, DM, upon request. Readers may apply for access to the de-identified transcripts by emailing the corresponding author at Douglas.McHugh@quinnipiac.edu.

Restrictions apply to the availability of these transcript data; disqualifying criteria for access consist of the following: data will not be available to any person under the age of 18 years old; and data will not be available to any person who currently is (or formerly was) a student, teacher, or employee of the schools from which participants in this study were recruited.

Zenodo: Contextualizing adolescents' self-awareness of problematic mobile phone use: a preliminary study - PUMP Survey and PUMP Raw Data. https://doi.org/10.5281/zenodo.4571013. ^29^

This project contains the following underlying data:

• Dodd PUMP Survey Raw Data.pdf
• Morgan PUMP Survey Raw Data.pdf

### Extended data

Zenodo: Contextualizing adolescents' self-awareness of problematic mobile phone use: a preliminary study - PUMP Survey and PUMP Raw Data. https://doi.org/10.5281/zenodo.4571013. ^29^

This project contains the following extended data:

• Karnaze PUMP Survey.docx

### Reporting guidelines

COREQ checklist for “Contextualizing adolescents’ self-awareness of problematic mobile phone use: a preliminary study”. https://doi.org/10.3886/E131701V2. ^30^

Data are available under the terms of the Creative Commons Attribution 4.0 International (CC-BY 4.0) License.
