# Supplementary material for: Contextualizing adolescents’ self-awareness of problematic mobile phone use: a preliminary study
Source: F1000Res. 2021 Aug 23;10:237. Originally published 2021 Mar 24. [Version 2] doi: 10.12688/f1000research.51339.2 (PMC11021880; doi:10.12688/f1000research.51339.2)
Supplement: Peer review history

# Reviewer report: Reviewer response for version 2

**DOI:** 10.5256/f1000research.76765.r265516

**Published:** 2024-04-30

**Authors:** Matea Bodrožić Selak

**Affiliations:** 1 Croatian Catholic University, Zagreb, Croatia

This study examined adolescents' perspectives on certain aspects of smartphone usage, specifically focusing on drivers of excessive smartphone use, the impact of smartphone usage on their relationships with family and friends, their sleep patterns, and the barriers or obstacles in their efforts to reduce mobile phone usage. A qualitative methodology was employed, allowing for a deeper insight and analysis of the researched phenomenon.

**Abstract**

 Clearly state the aim of the study.

**Introduction**

 In the introduction, it would be beneficial to present the theoretical perspective or framework within which the later results would be discussed and hypotheses formulated. Additionally, providing several examples of results from similar qualitative studies is necessary to contextualize the current research, and include studies that have examined self-awareness in adolescents.

 It is suggested to use the term "problematic smartphone use" throughout the paper instead of "addiction" because diagnostic measurement instruments are required to ascertain addiction and that is not the case in most previous research.

**Data collection**

 It might be beneficial to report the endpoints of the PUMP scale and indicate what a higher score on the scale signifies. Additionally, adding response frequencies (in the form of %) to the scale would provide insight into sample characteristics regarding problematic smartphone use.

 What are the exact questions discussed in focus groups? It is suggested to specify the exact questions posed in the focus groups that are relevant to the research questions in this study. Vrh obrasca

**Discussion**

 The discussion section also lacks a theoretical framework for interpreting the research findings. Additionally, it lacks citations of previous research and references to scientific literature, relying instead on YouTube videos. It is wise to situate the current study's results within existing theoretical frameworks to provide a deeper understanding of the implications and significance of the findings. For example, previous studies have explored various theoretical perspectives on problematic smartphone use, such as the uses and gratifications theory (Katz et al., 1973) [Ref 1], addiction models, and socio-cultural theories (neo-ecological theory, Navarro & Tudge, 2023) [Ref 2]. These frameworks offer valuable insights into the underlying mechanisms and socio-cultural factors driving excessive smartphone use and its impact on individuals and society.

**Limitations**

 One of the significant limitations that may be important to mention is the potential for participants to recall their mobile phone usage patterns accurately. According to some research, these behaviors are automatized and, consequently, inaccessible to consciousness (e.g., Oulasvirta et al., 2012) [Ref 3]. This limitation underscores the need for caution when interpreting self-reported data on smartphone usage, as participants may not accurately recall or represent their behaviors. To address this limitation, future research could incorporate objective measures of smartphone use, such as smartphone usage tracking apps or device usage logs, to complement self-report data for example. However, qualitative methods, such as interviews or focus groups, could provide deeper insights into participants' smartphone usage patterns and experiences, mitigating the impact of recall bias. By employing a combination of quantitative and qualitative approaches, researchers can enhance the validity and reliability of their findings, thereby advancing our understanding of problematic smartphone use.

**Conclusion**

 In the conclusion, it is important to summarise the main findings of the study.

Is the work clearly and accurately presented and does it cite the current literature?

Partly

If applicable, is the statistical analysis and its interpretation appropriate?

Partly

Are all the source data underlying the results available to ensure full reproducibility?

Partly

Is the study design appropriate and is the work technically sound?

Yes

Are the conclusions drawn adequately supported by the results?

Yes

Are sufficient details of methods and analysis provided to allow replication by others?

No

Reviewer Expertise:

developmental psychology, mental health

I confirm that I have read this submission and believe that I have an appropriate level of expertise to confirm that it is of an acceptable scientific standard, however I have significant reservations, as outlined above.

## References

[1] : Uses and Gratifications Research. *Public Opinion Quarterly* .1973;37(4) : 10.1086/268109 10.1086/268109

[2] : Technologizing Bronfenbrenner: Neo-ecological Theory. *Curr Psychol* .2022; 10.1007/s12144-022-02738-3 1-17 10.1007/s12144-022-02738-3 35095241 PMC8782219

[3] : Habits make smartphone use more pervasive. *Personal and Ubiquitous Computing* .2012;16(1) : 10.1007/s00779-011-0412-2 105-114 10.1007/s00779-011-0412-2

# Reviewer report: Reviewer response for version 2

**DOI:** 10.5256/f1000research.76765.r259388

**Published:** 2024-04-16

**Authors:** Marc Olivella-Cirici

**Affiliations:** 1 Health Services Research Group, Hospital del Mar Research Institute - IMIM, Barcelona, Catalonia, Spain; 2 CIBER of Epidemiology and Public Health, Madrid, Spain; 3 Pompeu Fabra University, Barcelona, Catalonia, Spain

The article addresses a subject of interest in public health, in constant and rapid change and which has a clear translation in the design of policies and regulations. The text follows a logical and easy-to-read narrative. The objective is well defined and fits both the design of the study and the research as a whole.

 Here are some suggestions for improvement:

 - The terminology is not consistent throughout the article (mobile phone; cell phone, cellular phone, smartphone...). I suggest homogenizing and using only the concept "smartphone" throughout the text. Therefore, the concept to be used would be "problematic smartphone use".

 - The theoretical perspective of the methodology suggests a socio-constructionist logic (Dew K., 2007 [Ref 1]) since the explanations and perceptions of the problematic uses of the smartphone are social and are constructed collectively (Olivella-Cirici M, et. al., 2022 [Ref 2]).

 - Which qualitative data analysis software was used? It would be interesting to reference it.

 - I'm not quite sure why the choice of this scale of problematic use of the smartphone (PUMP) is the most suitable. There are other scales that move away from the pathologizing optics of smartphone use that could also be of interest. It would be interesting to better justify the use of this scale.

 - I miss a better explanation of the link between the use of a survey, which is a quantitative analysis technique, and the use of a qualitative technique such as discussion groups. Why is this mixed methodology approach necessary? What does this add to the contextualization of adolescent self-awareness?

 - It would be interesting to attach the semi-structured script of the discussion groups as supplementary material.

 - The article was submitted for publication in early 2021, which is why the references are partially out of date. In a rapidly evolving field where app usage follows very specific trends, I suggest updating some citations in case a new version of the research is written.

 - The first half of the conclusion corresponds more to an introductory text of the article than to the conclusions derived from the research. I suggest focusing the conclusions on the results obtained and recommendations for future lines of research.

Is the work clearly and accurately presented and does it cite the current literature?

Partly

If applicable, is the statistical analysis and its interpretation appropriate?

Partly

Are all the source data underlying the results available to ensure full reproducibility?

Yes

Is the study design appropriate and is the work technically sound?

Yes

Are the conclusions drawn adequately supported by the results?

Partly

Are sufficient details of methods and analysis provided to allow replication by others?

Yes

Reviewer Expertise:

Public health; Uses of the Smartphone; Adolescent Health; Epidemiology; Health Inequalities; Mental Health.

I confirm that I have read this submission and believe that I have an appropriate level of expertise to confirm that it is of an acceptable scientific standard, however I have significant reservations, as outlined above.

## References

[1] : A health researcher's guide to qualitative methodologies. *Aust N Z J Public Health* .2007;31(5) : 10.1111/j.1753-6405.2007.00114.x 433-7 10.1111/j.1753-6405.2007.00114.x 17931290

[2] : Perceptions of the Uses of Cell Phones and Their Impact on the Health of Early Adolescents in Barcelona: a Qualitative Study. *Journal of Technology in Behavioral Science* .2022;7(2) : 10.1007/s41347-021-00228-0 130-140 10.1007/s41347-021-00228-0

# Reviewer report: Reviewer response for version 2

**DOI:** 10.5256/f1000research.76765.r92562

**Published:** 2021-09-06

**Authors:** Folashade Tawakalitu Alloh

**Affiliations:** 1 Department of Allied and Public health professions, University of East London, London, UK

The authors have addressed issues raised at the first round of review.

Is the work clearly and accurately presented and does it cite the current literature?

Partly

If applicable, is the statistical analysis and its interpretation appropriate?

Partly

Are all the source data underlying the results available to ensure full reproducibility?

Partly

Is the study design appropriate and is the work technically sound?

Yes

Are the conclusions drawn adequately supported by the results?

Yes

Are sufficient details of methods and analysis provided to allow replication by others?

No

Reviewer Expertise:

Mental health, qualitative research, quantitative research, minority health, health inequality.

I confirm that I have read this submission and believe that I have an appropriate level of expertise to confirm that it is of an acceptable scientific standard.

# Reviewer report: Reviewer response for version 1

**DOI:** 10.5256/f1000research.54496.r82119

**Published:** 2021-05-07

**Authors:** Marquisette Glass Lewis

**Affiliations:** 1 Health Sciences, Belhaven University- Atlanta, Atlanta, GA, USA

The manuscript was an easy read and written well. There are few things that I believe would be valuable.

1. What specific websites were mostly reviewed or downloaded from boys and girls?
2. When watching the films were the subjects talking or watching the movie?
3. What games were used the majority of the time?
4. How often did the subjects upgrade their phone and who purchased it?
5. Did any of the participants spend time looking up school work?
6. Did any of the participants have a house phone?

Is the work clearly and accurately presented and does it cite the current literature?

Yes

If applicable, is the statistical analysis and its interpretation appropriate?

Yes

Are all the source data underlying the results available to ensure full reproducibility?

Yes

Is the study design appropriate and is the work technically sound?

Yes

Are the conclusions drawn adequately supported by the results?

Yes

Are sufficient details of methods and analysis provided to allow replication by others?

Yes

Reviewer Expertise:

Healthcare, uterine artery embolization, HIV, uterine fibroids, COVID-19, diabetes, fibroids.

I confirm that I have read this submission and believe that I have an appropriate level of expertise to confirm that it is of an acceptable scientific standard, however I have significant reservations, as outlined above.

# Response

**Published:** 2021-08-12

**Authors:** Douglas McHugh

**Affiliations:** Quinnipiac University, USA

**Response to Reviewer - Marquisette Glass Lewis**

***Author response***: Thank you for your comments and feedback, and I wish to acknowledge the value of your contributions to improving this article. My co-authors and I are grateful for peer-reviewers who contribute to assuring that scholarship entering the field is of high quality and grounded in previous findings and appropriate methodologies. 

***Reviewer suggested revisions***:

 The manuscript was an easy read and written well. There are few things that I believe would be valuable.

 1.    What specific websites were mostly reviewed or downloaded from boys and girls?

 2.    When watching the films were the subjects talking or watching the movie?

 3.    What games were used the majority of the time?

 4.    How often did the subjects upgrade their phone and who purchased it?

 5.    Did any of the participants spend time looking up school work?

 6.    Did any of the participants have a house phone?

***Author response***: These are excellent questions that target the details of what specific content the adolescent participants were accessing on their smartphones as well as when and how often, and further contextualize smartphone upgrades and the presence of landline phones. Unfortunately, these questions are outside the scope of the data we collected for this preliminary study. Therefore, we will not be able to accommodate this requested revision simply because we do not have the relevant information. We agree that asking and gathering answers to these questions would be important to collect for future, generalizable quantitative studies. Thank you for this suggested improvement.

# Reviewer report: Reviewer response for version 1

**DOI:** 10.5256/f1000research.54496.r82756

**Published:** 2021-04-26

**Authors:** Folashade Tawakalitu Alloh

**Affiliations:** 1 Department of Allied and Public health professions, University of East London, London, UK

The manuscript explored the experiences of adolescence in managing smartphone use. This is an interesting and valuable research area in the context of the pandemic and mental health impact on adolescents. Qualitative data collection was gathered from adolescents using interpretive phenomenology to guide the research process.

**Introduction**

 A justification of why the study was implemented among students in the chosen schools can be helpful for readers. The authors mentioned reviewing the literature and the search terms used but has not clearly presented the outcome of the review or how this contributed to the study design.

**Method**

 What is the impact of watching the Youtube clip before discussion with participants? Authors did not clarify why this is needed and how it contributed to responses from participants.

 Developing themes, how is the data analysis phase influenced by phenomenology approach adopted for the study, this needs to be further discussed; how does this influence the findings?

**Results**

 Similarly, to the methods section, the result from the study should be presented within the framework of phenomenology approach.

**Discussion**

 A limitation of the study has been discussed due to the limited participants recruited for the study. However, the discussion can be improved by discussing the impact of smartphone use on mental health of adolescents.

Is the work clearly and accurately presented and does it cite the current literature?

Partly

If applicable, is the statistical analysis and its interpretation appropriate?

Partly

Are all the source data underlying the results available to ensure full reproducibility?

Partly

Is the study design appropriate and is the work technically sound?

Yes

Are the conclusions drawn adequately supported by the results?

Yes

Are sufficient details of methods and analysis provided to allow replication by others?

No

Reviewer Expertise:

Mental health, qualitative research, quantitative research, minority health, health inequality.

I confirm that I have read this submission and believe that I have an appropriate level of expertise to confirm that it is of an acceptable scientific standard, however I have significant reservations, as outlined above.

# Response

**Published:** 2021-08-18

**Authors:** Douglas McHugh

**Affiliations:** Quinnipiac University, USA

**Response to Reviewer - Folashade Tawakalitu Alloh**

*Author response*: Thank you for your comments and feedback. I wish to acknowledge the value of your contributions to improving this article. My co-authors and I are grateful for peer-reviewers who contribute to assuring that scholarship entering the field is of high quality and grounded in previous findings and appropriate methodologies. 

***Reviewer suggested revisions***:

**The manuscript explored the experiences of adolescence in managing smartphone use. This is an interesting and valuable research area in the context of the pandemic and mental health impact on adolescents. Qualitative data collection was gathered from adolescents using interpretive phenomenology to guide the research process.**

***Introduction***

**1. A justification of why the study was implemented among students in the chosen schools can be helpful for readers.**

*Author response*: Thank you for this prompt to clarify why we targeted adolescents in this study. We wrote in the introduction the following to draw readers’ attention to research on problematic mobile phone use that has involved the general population and why adolescents in particular represent a vulnerable age group,

 “Among the general population, determinants and effects of problematic phone use have been studied, screening tools developed, and prevalence of mobile dependency measured. [2,4-5] While the detrimental effects of problematic mobile phone use on sleep, stress, anxiety, and depression can occur at various ages [2], adolescents comprise a notably vulnerable age group. This relates to decreased levels of self-control and low resistance to peer pressure. Steinberg and Monahan (2007) noted “susceptibility to peer pressure in adolescence follows an inverted U-shaped curve, increasing during early adolescence, peaking around age 14, and declining thereafter.” [6]

 This together with the findings of our literature review of problematic mobile phone use is the justification for our focus on 13-15 year-olds as study participants. 

**2. The authors mentioned reviewing the literature and the search terms used but has not clearly presented the outcome of the review or how this contributed to the study design.**

*Author response*: We conducted a comprehensive review of the literature pertaining to adolescents and problematic mobile phone use in order to make readers of aware of what was available and to highlight the lack of qualitative data for those aged 13-15. The second paragraph of the introduction describes both how we conducted the literature review and a succinct summary of its findings (highlighted in bold below), consistent with the Brief Report category of F1000 Research. The lack of qualitative data for adolescents informed our choice to pursue a qualitative research question and study design that sought to explore the context of problematic mobile phone use with 13-15 year-olds..

 “We searched the PubMed database using the query “((Smartphone OR mobile phone OR cell phone) AND (dependency OR dependence OR problematic)) AND (adolescent OR adolescence OR teenage OR youth)” **and found with regard to adolescents that studies are limited to reports of prevalence and associations with adverse wellness. More specifically: cross-sectional studies have measured prevalence of mobile phone dependency [1,7-8]; mobile phone dependence correlated positively with unintentional injuries (odds ratio = 1.452) [9]; depressive symptoms, higher interpersonal anxiety, and lower self-esteem have been correlated with problematic cell phone use [10-12]; and Walsh et al (2008) held qualitative focus groups with Australian 16-24 year-olds and found extreme attachment to phones with symptoms of behavioral addiction evident in participants' descriptions. [13] Adolescent-specific investigations are limited to mobile phone dependency prevalence [1,7]; and to correlation with depression, interpersonal anxiety, lower self-esteem, and symptoms of behavioral addiction evident. [10,13] Adolescents are knowledgeable agents capable of representing and articulating the different aspects of how they interact with smartphones. Yet little work has been devoted to exploring how adolescents experience mobile phone dependency and what meanings and interpretations they attach to it.**” 

***Method***

**3. What is the impact of watching the Youtube clip before discussion with participants? Authors did not clarify why this is needed and how it contributed to responses from participants.**

*Author response*: In short, this was to help elicit participants’ views on Snapstreaks. It is not uncommon in humanities or sociology qualitative research for researchers to employ the use of artifacts (i.e., something created by humans which gives information) for participants to react or respond to (Crotty, 1998; Edwards et al, 2015). These artifacts are used, along with an episodic semi-structured interviewing style, to elicit participants’ views about the subject under investigation. The nature of this type of research is inherently subjective in contrast to basic science or clinical research where data can be objectively measured. In our case, the artifact was a YouTube-hosted CBS News interview with Tristan Harris the CEO of the social media app Snapchat which uses Snapstreaks. The adolescent participants are knowledgeable agents capable of representing and articulating what they think and believe (or don’t think or don’t believe) about the content of the video. In so doing, we as researchers were able to better explore from the qualitative data collected, how the use of social media apps may increase excessive mobile phone use in adolescents. Our citation of the video means that readers of the article can watch it and make their own judgments.

 We have added the following to the Data Collection and Analysis section of the Methods to clarify our rationale:

 “This video clip artifact was employed to help elicit participants’ views on Snapstreaks. It is not uncommon in humanities or sociology qualitative research for researchers to employ the use of artifacts (i.e., something created by humans which gives information) for participants to react or respond to (Crotty, 1998; Edwards et al, 2015). These artifacts are used, along with an episodic semi-structured interviewing style, to elicit participants’ views about the subject under investigation.”

 References: 

 Edwards R, Fenwick T, Sawchuk P. Emerging Approaches to Educational Research: Tracing the Socio-Material. London: Routledge; 2015

 Crotty M. The foundations of social research: meaning and perspective in the research process. London: Sage Publications, 1998

**4. Developing themes, how is the data analysis phase influenced by phenomenology approach adopted for the study, this needs to be further discussed; how does this influence the findings?**

*Author response*: Thank you for prompting us to better describe our data analysis processes in relation to phenomenology.We have added the following to the Data Collection and Analysis section of the Methods (new content highlighted in bold):

 “ **The primary goal of researchers during the data analysis process in a phenomenological study is to deduce meaning out of the themes identified through organizing the data, coding the data, deducing categories, identifying common themes, making interpretations, and maintaining a reflective journal. The latter is an important step on phenomenological study to address researcher bias and overall credibility. It involves maintaining field notes detailing researchers’ choices and decision-making; it serves to keep the themes generation and interpretation transparent, coherent, and simple (Chenai 2011). **K.G. wrote field notes after each focus group to reflect on the participants’ observations, K.G.’s growing insights, and how her facilitator-outsider role informed the research process. [16] Audio recordings were transcribed verbatim then subject to de-identified inductive qualitative analysis using a constant comparison approach. [17] De-identified transcripts were read, then hand-coded by assigning first-stage preliminary codes. A.K. and D.M. then met together to group similar codes into categories and identify major concepts while referencing K.G.’s memos and field notes. Iteratively, major concepts were further organized and expressed as themes and subthemes.”

***Results***

**5. Similarly, to the methods section, the result from the study should be presented within the framework of phenomenology approach.**

*Author response*: We have added the following to the Qualitative Findings section of the Results to make more explicit the connection between our research approach and the presentation of our findings (new content highlighted in bold). The results of data explication of phenomenological studies is ultimately theme generation and interpretation of the participants’ experiences of the phenomenon being investigated:

 “ **Phenomenological data explication involves researchers’ inferring the meaning behind participants’ words and articulating experiences through thematic statements (Bassett 2004)**. Participants possessed a notable awareness of problematic smartphone use in themselves and their peers. Four main themes with associated subthemes emerged **from our phenomenological data analysis processes**: drivers of excessive smartphone use, with family or friends, barriers to healthier smartphone use, and nighttime habits; Illustrative quotes are identified by anonymous participant codes.”

***Discussion***

**6. A limitation of the study has been discussed due to the limited participants recruited for the study. However, the discussion can be improved by discussing the impact of smartphone use on mental health of adolescents.**

*Author response*: Thank you for this welcome recommendation to include a short discussion of the impact of problematic smartphone use on adolescents’ mental well-being. We have added the following to the end of the first paragraph in the Discussion section:

 “Problematic smartphone use has been negatively associated with adolescents’ mental well-being. Souza Pereiva et al (2020), in a study of 359 female and 308 male students aged 13-18 years, associated it adversely with physical activity levels, personal interactions, academic performance, and mood disturbances. Abi-Jaoude et al (2020) also report, “Evidence from a variety of cross-sectional, longitudinal and empirical studies implicate smartphone and social media use in the increase in mental distress, self-injurious behaviour and suicidality among youth; there is a dose–response relationship, and the effects appear to be greatest among girls.” As we also noted from our participants’ responses, Abi-Jaoude et al describe smartphone app-facilitated social comparisons among adolescents that negatively affected their self-view and led to increased cyberbullying and promotion of self-harming behaviors. They call for an increase in public awareness of problematic smartphone use. The contexts highlighted by our study may help inform social policy interactions aimed at promoting safe, inclusive, and nourishing home or educational environments.”
